# A foreign body in inguinal canal: A case report

**DOI:** 10.1016/j.ijscr.2018.08.026

**Published:** 2018-08-19

**Authors:** Amer Hashim Al Ani, Mohammad Bakri Hammami, Obaidah M. Mukhles Adi

**Affiliations:** a Department of General Surgery, Sheikh Khalifa Medical City, Ajman, United Arab Emirates; b College of Medicine, University of Sharjah, Sharjah, United Arab Emirates

**Keywords:** Retained item, Inguinal canal, Complications, Foreign body, Case study

## Abstract

**Introduction:**

Inguinal bladder hernias are rare incidents accounting for 1–3% of all inguinal hernia. Most of those cases are discovered intraoperatively accounting for the high incidence of bladder injuries during the repair. Symptoms can be variable depending on the size of the herniated bladder.

**Presentation of case:**

We present here a 70 years old obese male patient, with history of CVA & on regular Aspirin ingestion. He presented to the ER as a case of intestinal obstruction due to suspicion of strangulated inguinal hernia based on an unclear Ultrasound picture. He underwent an emergency exploration of the hernia where the balloon of a Foley’s catheter was found inside a diverticulum of the urinary bladder, herniated through an Ogilvie hernia.

**Discussion:**

Ogilvie hernia is a rare incident that happens mostly in elderly males. The herniated part contains prevesical fat, bladder andor loops of intestines. Diagnosis must be made preoperatively through CT scan or Cystourethrogram. First line management involves surgical reduction of the bladder and hernia repair. Resection of the herniated part is generally limited to necrotic tissues.

**Conclusion:**

Surgeons must have high index of suspicion for elderly obese males with inguinal hernias and urinary or intestinal obstruction symptoms. Careful preoperative planning must be made to avoid repair associated bladder injuries.

## Highlights

• Bladder hernias are rare incidents that are mostly discovered intraoperatively.
• Diagnosis must be made preoperatively to avoid risk of injuring the bladder.
• High index of suspicion for bladder hernia is vital in elderly males with urinary or intestinal symptoms.

## 1 Introduction

Inguinal bladder hernia is a rare clinical condition that accounts for 1–3% of all inguinal hernias [1]. Higher incidence has been reported in obese men older than 50 years which reaches up to 10%. Inguinal bladder hernia was first described by Levine in 1951 as “scrotal cystocele” [2]. It is generally caused by increased abdominal pressure or a defect in the peritoneum which classifies it as a direct inguinal hernia and explains the increased incidence with age [3].

Ogilvie’s inguinal hernia, which we present here, is caused by a small defect in the medial part of conjoint tendon and above the pubic tubercle. It usually contains prevesical fat and part of urinary bladder [4,5]. It can involve variable parts ranging from a small diverticulum to a whole bladder and ureter [2].

Despite the advancement in diagnostic technologies and since most of the cases are asymptomatic, it is still a challenge to diagnose inguinal bladder hernia where <7% is diagnosed preoperatively and many of the cases are identified incidentally [3].

Patients with complete bladder herniation will present with double voiding which means the patient should compress his scrotum to fully evacuate the bladder [6]. Inguinal bladder hernias are associated with many complications leading to obstructive uropathy, urinary tract infection, bladder infarction, epididymitis or even cancer. moreover, the risk of accidental bladder injury during repair is 12% [3].

In this prospective, single Centre, Case report study, we present a case of 74-year-old male who presented with sliding direct Ogilvie inguinal hernia and upon surgical exploration a Foley’s catheter was found in the herniated part of the bladder. The case was managed in Al Bashir teaching hospital in Amman, Jordan in 2010 and was followed up in the same hospital. This paper was written according to SCARE criteria for case reports [8].

## 2 Case presentation

A 74-year-old obese male was presented to ED (Emergency Department) with abdominal pain, distension, vomiting and diarrhea for 5 days. His past medical history is significant for hypertension and CVA (Cerebrovascular accident) 3 years before admission. He had a Colonic polyp removed 5 years ago. In addition, He had a history of gallstones removed 10 years ago. He was on Atenolol. Captopril and, Aspirin. On examination, the patient had generalized weakness of the left side of his body due to previous CVA.

There was a tender irreducible swelling in the right inguinal region, covered by normal skin. Since the patient had a history of stroke years ago, it was not possible to ask him about duration of the swelling, pain at the site, or to cough to evaluate the swelling more. The vital signs were within normal. Bowel sounds were sluggish. Digital rectal examination showed nothing of significance.

Lab results showed Blood Urea: 11.8 mmol/L, Serum Creatinine: 116.96 μmol/L, Serum Potassium: 5.5 mmol/L, Blood Sugar: 10.3 mmol/L, WBC Count: 14.2 × 10^3^/mm^3^, Hemoglobin: 12.4 g/dL, Platelets: 312 × 10^3^/mm^3^.

**Fig. 1:**
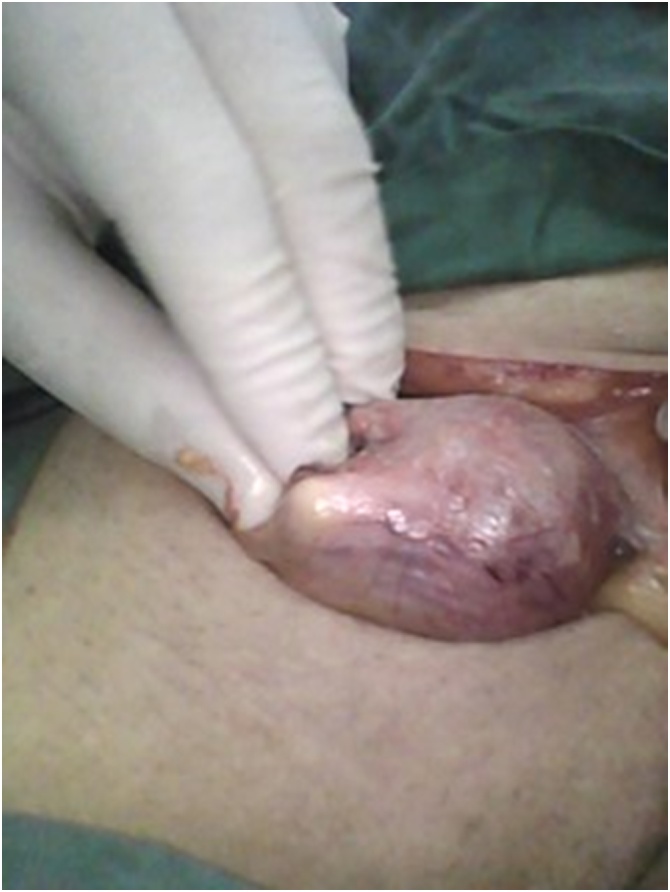
Sliding Ogilvie hernia showing foreign body during the exploration of hernia.

**Fig. 2:**
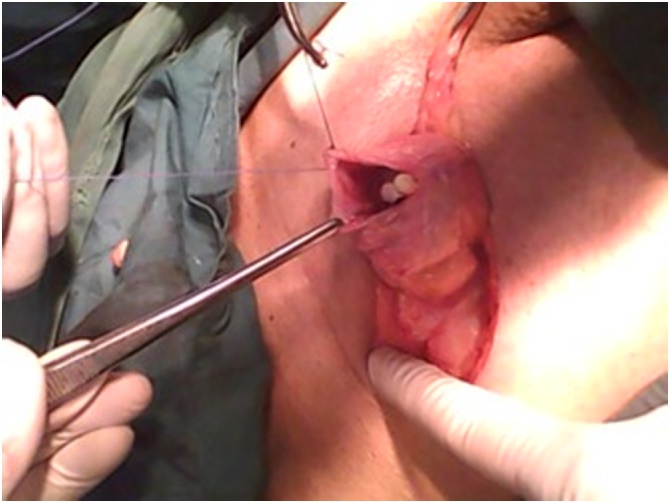
Balloon of a Foley’s catheter within the sliding part of the bladder in the inguinal hernia.

On Abdominal ultrasound, the gallbladder wasn’t visualized, but a cystic like lesion in the right inguinal region mostly representing a bowel loop was seen. The tentative diagnosis for this case was strangulated right inguinal hernia causing intestinal obstruction. Consent was taken from the patient’s next of kin and the right inguinal region was explored. A sliding direct Ogilvie inguinal hernia was discovered. There was a well circumscribed soft mass of a narrow neck, protruding from the posterior wall of the hernia that looks like a foreign body (Fig. 1). On exploration, the mass was the balloon of a Foley's catheter in the sliding part of the bladder within the inguinal hernia, and the urinary bladder was part of the posterior wall of the inguinal canal (Fig. 2). The direct sliding Ogilvie inguinal hernia was repaired, and Explorative laparotomy was done to deal with the cause of intestinal obstruction through a mid-line incision. Upon exploration, a superior mesenteric artery occlusion was noticed causing strangulation of all small bowel (excluding first 100 cm of jejunum), all right colon and most of transverse colon. Resection of all gangrenous bowel was done, with end to end jejuno-colonic anastomosis. The patient’s case deteriorated over the next few days until he died on the 5th day post operation due to cardiac issue.

## 3 Discussion

Studies have shown that Bladder hernia is uncommon to happen, constituting only 1–3% of all inguinal hernias [1,9]. The main risk factors are male sex, obesity and higher age [1] which were all present in our case. Many case reports and case series described the presentation of bladder hernias in different sizes. However, no previous study described Foley’s catheter within the herniated bladder.

Ogilvie hernia is a much rarer type of hernias which usually occurs in elderly males, as in our case. It’s characterized by a narrow short neck. Prevesical fat, portion of the bladder andor intestines usually are found herniating through a defect in the medial part of the conjoint tendon, just above the pubic tubercle. No epidemiological data about this type is reported in literature [4,5].

The presence of urinary symptoms depends on the size of the herniated part of the bladder. [7] Most of those cases show small hernias that are generally asymptomatic. Larger hernias can cause Lower Urinary Tract Symptoms (LUTS) like frequency, urgency, nocturia, hematuria and superimposed infections. Severe bladder herniation usually presents with 2-stage micturition, where patients need to squeeze the hernia in order to complete voiding [10]. In our case, the patient had symptoms of intestinal obstruction and no urinary symptoms. Although the herniated part of the bladder was small, the presence of the Foley’s catheter within the herniated part showed an irregular pattern on US, which, based on the clinical picture, was thought to be strangulated loops of intestines.

Less than 7% of all Foley’s catheter cases are diagnosed preoperatively, 16% are diagnosed postoperatively due to the complications, while 77% of the cases, including our case, are diagnosed intraoperatively. The importance of diagnosing bladder hernias before the operation lies in the high risk of damaging the bladder (12%) during the repair, which is significantly reduced if preoperative diagnosis was made [1].

Gold standard imaging modality for diagnosis of Bladder hernias is Cystourethrogram. This modality is often used to assess the cases of outlet obstruction, which might or might not be seen in cases of bladder hernias. [7,11]. Ultrasound use for bladder hernias is controversial in previous literature. It’s particularly helpful for screening in Upper urinary tract, but it has limited validity in bladder hernias [3,11,12]. Ultrasound in our case was not helpful in diagnosis due to the small size of the hernia and the presence of the Foley’s catheter. An alternative modality could be the Computed Tomography (CT scan) which adds detailed information that assists in preoperative planning of these cases. [2]. In our case, there were no urinary symptoms that could indicate cystourethrogram. Also, CT scan was contraindicated based on the patient’s Urea and Creatinine levels.

Bladder hernias can be managed conservatively by catheterization and decompression of the bladder [3] or by surgical approach which can be open or laparoscopic. Open surgical approach with or without a mesh is still the preferred method in literature [2].

Surgery involves resecting or reducing the herniated part of the bladder. Reduction is generally preferred to preserve the bladder volume and to prevent the need to incise and suture the bladder increasing the risk of infection. Resection is performed only in cases of necrotic herniated parts of bladder, small hernia neck (<0.5 cm) and bladder tumor [1,11]. In our case, the herniated part of the bladder was reduced.

## 4 Conclusion

Inguinal Bladder Hernias are rare incidents commonly missed preoperatively. The symptoms can be variable depending on the size of the herniated part of the bladder. Surgeons must have high index of suspicion for elderly obese males with inguinal hernias and urinary or intestinal obstruction symptoms. Diagnosis must be made through imaging modalities and careful preoperative planning must be carried out to prevent bladder injuries.

## Conflicts of interest

Author and Co-authors have nothing to disclose.

## Sources of funding

This study was not funded by any sponsor.

## Ethical approval

This study was exempted from ethical approval in Al Bashir teaching Hospital Amman. Jordan.

## Consent

A written informed consent was obtained from the patient’s next of kin. Copy of the written consent is available for review by the Editor-in-Chief of this journal on request.

## Author contribution

Study design: Amer Hashim Al Ani.

Acquisition of data: Amer Hashim Al Ani.

Analysis and interpretations: Amer Hashim Al Ani, Mohammad Bakri Hammami, Obaidah Adi.

Manuscript draft: Mohammad Bakri Hammami, Obaidah Adi.

Revision: Amer Hashim Al Ani.

All authors have approved the final article.

## Registration of research studies

Registration Date : 09:09 March 11, 2018.

Research Registry UIN: researchregistry3807.

Primary Investigator and Sponsor Public and Scientific : Amer Al Ani.

Public and scientific title of research : Foreign bodies in inguinal canal canal.

## Guarantor

Dr. Amer Hashim Al Ani.
